# Web-Based COPD Risk Self-Assessment Identifies a High-Risk Group With HRQoL Resembling Self-Reported COPD: Cross-Sectional Survey

**DOI:** 10.1177/00469580261432827

**Published:** 2026-04-08

**Authors:** Bernt Bøgvald Aarli, Kjell Garatun-Tjeldstø, Odd Erik Johansen, Laxmi Bhatta, Arnt-Ove Hovden, Oeystein Naess, Marthe Gundersen, Bjarte Nore

**Affiliations:** 1 University of Bergen, Norway; 2 Haukeland University Hospital, Bergen, Norway; 3 KBB Medic AS, Bergen, Norway; 4 JOE Medical, Hövik, Norway; 5 NordicRWE, Oslo, Norway; 6 Astra Zeneca, Oslo, Norway; 7 LHL, Oslo, Norway; 8 Bergen Legevakt, Norway

**Keywords:** COPD, web based, self-assessment, algorithm-based, health-related quality of life, breathlessness, risk factors

## Abstract

This study aimed to evaluate a web-based self-assessment tool for COPD case finding using algorithm-based COPD risk stratification, and to compare breathlessness (mMRC) and respiratory health-related quality of life (HRQoL) across risk groups and with self-reported COPD. We conducted a cross-sectional web-based survey of anonymous visitors completing a web-based self-assessment tool including demographics, COPD risk factors, breathlessness (mMRC), and respiratory HRQoL (CAT and K-BILD). Only respondents with complete responses were analyzed. COPD risk was algorithmically classified as high- or low-risk based on responses to age, smoking or dust exposure, respiratory symptoms, history of exacerbations, and the mMRC score. Individuals classified as having a high-risk of COPD received a prompt recommendation to consult their general practitioners. Group comparisons were performed using chi-square or ANCOVA, adjusting for covariates (age, sex, and current smoking). All analyses were exploratory, without corrections for multiple testing. In total, 57 847 people visited the website between Oct 2018 and Nov 2024, of whom 8779 (63% female) completed the full survey. Of those 8779 (63% female), 799 reported a COPD diagnosis (mean age 64.1 years). The high-risk (n = 6569) and the low-risk (n = 1411) groups were younger than the self-reported COPD group (58.4, and 54.6 vs 60.1 years, *P* < .0001). Self-reported COPD was associated with a lower proportion of never smokers than the high- and low-risk groups (7.1% vs 15.0% and 22.0%, respectively; *P* < .0001). Individuals with self-reported COPD had greater breathlessness (mMRC: 1.74 ± 1.28, 1.39 ± 1.0 and 0 ± 0) and poorer HRQoL (CAT 18.9 ± 7.9, 15.9 ± 8.0 and 10.6 ± 5.5; K-BILD 64.4 ± 19.2, 73.1 ± 18.6 and 87.4 ± 12.8) than both high- and low-risk groups (all *P* < .0001). Breathlessness and respiratory HRQoL differed across algorithm-based COPD risk stratification groups, and the algorithmically identified high-risk group showed a profile closer to self-reported COPD than the low-risk group. These findings support a web-based self-assessment tool as an approach for early COPD case finding and identifying undiagnosed COPD, although further validation is required.

## Introduction

Chronic obstructive pulmonary disease (COPD) is considered a partly preventable and modifiable condition characterized by irreversible or poorly reversible airflow obstruction and persistent respiratory symptoms due to airway and/or alveolar abnormalities.^1^ In 2021, it caused approximately 3.5 million deaths (5% of all global deaths), with nearly 90% of those deaths in people under 70 years of age in low- and middle-income countries.^2^ The prevalence of COPD globally has been reported with different numbers, depending on classification criterion used, with prevalence in some studies as high as 12.6% in people with age over 40 years.^3^ Population-based studies of individuals aged >40 years have estimated the prevalence of COPD to be 6% to 7%.^4,5^ However, a significant proportion are believed to be undiagnosed.^6^

Presently, in several guidelines, active screening using spirometry is not recommended, as it has not been associated with improvements in morbidity or mortality (eg, as discussed by the United States Preventive Service Task Force).^7^ However, it is recommended to perform active case finding, that is, implement a strategy targeting early identification of people at high risk for COPD, for example, by using a questionnaire.^8^ Early identification of individuals at risk for COPD, based on respiratory symptoms and exposure to smoking or dust, may enable timely prevention, diagnosis, and treatment, potentially reducing COPD-related morbidity, mortality, and associated individual and societal health costs. COPD is included in the World Health Organization (WHO) Global Action Plan for the Prevention and Control of Noncommunicable Diseases (NCDs) and the United Nations 2030 Agenda for Sustainable Development.^2^

One potential approach to facilitate the early identification of individuals at high risk of COPD (ie, active case-finding) is the use of a web-based self-assessment tool. In this setup, an algorithm evaluates user responses to specific questions and standardized questionnaires to identify individuals with a higher likelihood of having COPD. If the assessment indicates possible COPD, the tool can generate a prompt, encouraging the individual to consult a physician for further evaluation, potentially supporting earlier detection and intervention. This was previously demonstrated in the COPD Assessment in Primary Care to Identify Undiagnosed Respiratory Disease and Exacerbation Risk (CAPTURE) study, developed with support from the National Heart, Lung, and Blood Institute, part of the National Institutes of Health in the US. Researchers found that CAPTURE successfully identified almost half of the participants who had moderate to severe forms of previously undiagnosed COPD. The algorithm used in CAPTURE was based on participants’ responses to 5 questions concerning breathing difficulties, exposure to chemicals, and air pollution.^9^

In this study, we evaluated the applicability of a novel web-based tool (in Norwegian) that is freely available on public websites in Norway. The tool used selected questions along with 3 respiratory questionnaires to assess the risk of COPD.

## Material and Methods

This cross-sectional survey collected responses from visitors to a public freely accessible Norwegian website available at 2 domains: on the website of the Norwegian Heart and Lung Association (LHL), a national patient advocacy organization (https://www.lhl.no/kampanje/jeg-har-kols/) and https://kolshjelpen.no/. The web-based self-assessment tool was launched in connection with World COPD Day 2018 and remained freely accessible on public websites thereafter, reflecting real-world availability rather than a predefined recruitment period. During the first 2 weeks, the survey was conducted through a Facebook campaign targeting Norwegians aged 35 years and older. Following the initial campaign period, the website remained openly accessible via the homepage of the LHL. Given the exploratory nature of the study, no a priori sample size calculation was performed.

No identifiable data were collected from the respondents, such as IP-addresses, e-mail addresses, cookies, or browser history, and all information was submitted anonymously. The survey responses were stored on a domestic web server (ServeThe World AS).

Our survey, which involved anonymized data, no identifiable personal data, no intervention, and no collection of biological material, required no written informed consent, in line with the guidelines of the Regional Committees for Medical and Health Research Ethics (REK) in Norway that were consulted. Thus, formal ethical approval was not sought, as the study did not fall under their mandate. This aligns with the principles of the Declaration of Helsinki, that recognizes the role of national ethical standards in assessing risk and necessity for such measures. All potential participants were exposed to a paragraph at the beginning of the survey, highlighting that the data collection followed the general data protection regulation (GDPR). The study was conducted and reported in line with the STROBE guidelines (see online material).

### Questions and Questionnaires

This website offered visitors the opportunity to complete a brief self-assessment of COPD risk, provided that they had not previously reported a COPD diagnosis. Participants were also asked to provide basic demographic information and complete 3 respiratory questionnaires. Individuals who had indicated a prior COPD diagnosis were invited to answer the same questions and questionnaires; however, their responses were not evaluated for COPD risk (see online material for a translated version).

There was an initial explanation of the intent with the survey, thereafter, respondents would move on to provide demographic and background data including gender (female, male, not reported), age, smoking status (“never,” “former,” “current”) and knowledge about having been tested for α1-antitrypsin deficiency (“yes – confirmed,” “yes – confirmed normal,” “no – not tested,” “do not know”). The self-risk COPD assessment questions were written in Norwegian, and comprised 3 initial questions (“Are you older than 35 years of age?”: yes/no; “Have you been exposed to smoke or dust?”: yes/no, and “Do you have chronic respiratory symptoms?”: yes/no), which was followed by 2 additional questions if 1 of the 3 initial questions was positive; 1 was related to respiratory exacerbations (“Have you experienced respiratory exacerbations?”: Never/Once/Twice/3 times or more), and the other was an adapted Modified Medical Research Council dyspnea scale (mMRC).^10,11^ The mMRC dyspnea scale is simple to administer and is used for grading the effect of breathlessness (dyspnea) on daily activities. It measures perceived respiratory disability, allowing patients to indicate the degree to which dyspnea symptoms affect their daily functioning. Participants selected 1 of the 5 statements that most closely corresponded to their degree of breathlessness related to activities (“1”: only with strenuous exercise, “2”: when walking up a slight hill, “3”: when walking on level ground, “4”: stops for breath after walking about 100 m, and “5”: breathless when dressing or undressing).

This self-assessment provided one of three feedbacks (translated from Norwegian) to the respondents: “very low likelihood of COPD”, “low likelihood of COPD,” and “moderate to high risk of COPD – consider consulting a physician.” For the purpose of this analysis, individuals receiving the first two messages were categorized as having a low risk of COPD, whereas those receiving the third message were categorized as having a high risk for COPD.

The algorithm classified respondents as high-risk for COPD if all of the following criteria were met: age ≥ 35 years, self-reported exposure to smoke or dust (answered “yes”), presence of chronic respiratory symptoms (answered “yes”), and history of respiratory exacerbations (>0), or a modified Medical Research Council (mMRC) dyspnea score > 0.

Respondents were classified as having a low risk for COPD if they met any of the following conditions: age < 35 years, OR Age ≥ 35 years, no exposure to smoke or dust, and no chronic respiratory symptoms, OR Age ≥ 35 years, no chronic respiratory symptoms, no history of respiratory exacerbations, and mMRC = 0.

All respondents were then invited to complete 2 additional questionnaires: the COPD assessment test (CAT) and The King’s Brief Interstitial Lung Disease questionnaire (K-BILD). CAT^12^ was developed in 2009 to measure the impact of COPD on Health-related Quality of Life (HRQoL) and aid patient-physician communication. This patient-completed questionnaire is straightforward and easy to administer and is designed for routine use in clinical practice. It takes approximately 2 min to complete and capture the responder’s current condition. The 8 items included assess cough, phlegm, chest tightness, breathlessness going up hills and stairs, activity limitations at home, confidence leaving home, sleep, and energy. The sum score spans from 0 to 40, with a higher score indicating worse HRQoL. It has a broad coverage of the impact of COPD on the patient’s daily life. It has also been applied to patient groups other than those with COPD, such as interstitial lung disease (ILD) and post-COVID.^13,14^ The Norwegian validated version of the CAT was used in this survey.

The K-BILD is a validated instrument specifically designed to assess HRQL among patients with ILD.^15^ It comprise 15 questions scored from 0 to 7, with different dimensions: psychological (7), breathlessness and activities (4), chest symptoms (3), and financial status (1). Higher scores indicate better HRQL, with a range is 0 to 100. K-BILD has been validated in Norwegian^16^ and has also been used outside the context of ILD (like in long-term survivors of esophageal cancer).^17^ Although the K-BILD was originally developed for interstitial lung disease, it was included in this study as a complementary measure of broader health-related quality of life. Unlike CAT and mMRC, which primarily capture COPD-specific symptoms and breathlessness, K-BILD also assesses psychological and functional domains. This allowed us to explore whether individuals identified as high risk for COPD demonstrate an HRQoL profile resembling that of self-reported COPD beyond symptom burden alone.

Participants were allowed to discontinue the questionnaire at predefined points and proceed directly to the recommendations page (“Skip to recommendations”), resulting in several incomplete responses by design.

### Data Analysis

The analysis includes all respondents with complete data for the respiratory-related questionnaires collected during the defined study period. Categorical variables are presented as n (%) and continuous variables as mean (SD) or mean (SE). The primary exploratory analysis compared group scores for CAT, mMRC, and K-BILD using ANCOVA, adjusting for age, gender, and current smoking status. Analyses were performed both in the overall sample and within subgroups defined by smoking status.

Additional analysis compared baseline characteristics, and responses to the specific K-BILD question 15 about financial implications. These comparisons were performed with chi-squared test or ANCOVA (adjusting for age, gender and current smoking).

Spearman’s rank correlation coefficients were additionally used to assess univariate relationships between the CAT and responses to the mMRC, K-BILD, and the K-BILD item addressing financial burden due to the lung condition, in the overall sample.

## Results

**Figure 1.**
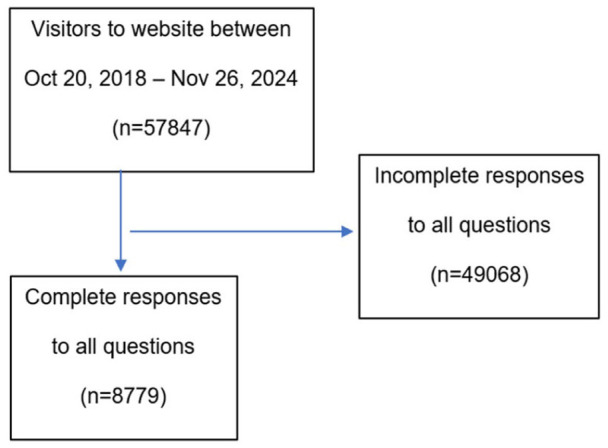
Visitor flow and completeness of responses (October 29, 2018-November 26, 2024).

Between October 28, 2018, and November 26, 2024, a total of 57 847 visitors accessed the website, of whom 8779 provided complete responses and were included in the analysis (Figure 1). A total of 799 respondents (9.1%) self-identified as having COPD. Of the remaining respondents 6569 (74.8%) were algorithmically classified as being at high-risk for COPD, and 1411 (16.1%) were classified as being at low risk. Table 1 depicts the characteristics of the respondents across groups, and the majority self-identified as females (57.4%-64.3%). There was a clear age-gradient, with a more advanced age reported by those with established COPD (mean age 64.1 years) compared with the high-risk group (58.4 years) and the low-risk group (54.6 years). A similar gradient was observed for former smoking, with a higher proportion among individuals with self-reported COPD (74.8%) than among those at high risk (50.2%) and low risk (43.8%). In contrast, current smoking was less common among individuals with self-reported COPD (18.1%) than among those classified as high risk (34.8%) or low risk (34.2%). Overall, few respondents reported having been tested for α1-antitrypsin deficiency. Specifically, 3.2% of individuals with self-reported COPD, 2.2% of those classified as high risk, and 0.6% of those classified as low risk reported having undergone testing. Only 1.8% of the individuals with established COPD group confirming this, compared to 1.1% in the high-risk group and 0.1% in the low-risk group. Most of the participants did not provide information on this item or were unaware of whether they had been tested.

**Table 1.** Self-reported Characteristics Across COPD Risk Groups in Respondents with Complete Responses to All Questionnaires (n = 8779).

Variables	Self-declared COPD	High-risk for COPD	Low-risk for COPD	Statistics
N	799	6569	1411	
Age, years	64.1 (9.7)	58.4 (11.3)	54.6 (11.3)	*P* < .0001
Sex				
Male (M)	42.6% (340)	35.7% (2342)	40.5% (571)	*P* < .0001
Female (F)	57.4% (459)	64.3% (4227)	59.5% (840)	
Smoking				
Never	−7% (56)	−15.0% (986)	−22.0% (310)	*P* < .0001
Current	−18.1% (145)	−34.8% (2284)	−34.2% (483)	
Former	−74.8% (598)	−50.2% (3299)	−43.8% (618)	
α1-antitrypsin deficiency				
Confirmed deficiency	−1.8% (14)	−1.1% (74)	−0.1%/2	*P* < .001
Confirmed normal	−1.4% (11)	−1.1% (71)	−0.5% (7)	
Not tested	−19.9% (159)	−18.8% (1235)	−14.6% (206)	
Do not know	−8.6% (69)	−4.7% (378)	−4.7% (378)	
Missing	−68.3% (546)	−73.9% (4855)	−81.6% (1152)	
Experienced respiratory exacerbations?				
Never	−56.4% (451)	−68.6% (4507)	−100%/1411	*P* < .0001
Once	−19.1% (153)	−16.8% (1100)	−0 (0%)	
≥Twice	−24.4% (195)	−14.6% (962)	−0 (0%)	
mMRC	1.74 (1.28)	1.39 (0.98)	0 (0)	*P* < .0001
CAT	18.9 (7.91)	15.9 (7.98)	10.6 (5.54)	*P* < .0001
K-BILD	64.4 (19.2)	73.1 (18.6)	87.4 (12.8)	*P* < .0001
K-BILD Q 15: Are you financially worse off because of your lung condition?	5.4 (1.7)	6.1 (1.5)	6.7 (1.4)	*P* < .0001

*Note.* Data are presented as mean (SD) or % (n). Comparisons across groups were conducted using analysis of variance or the chi-square test.

COPD = chronic obstructive pulmonary disease; mMRC = Modified Medical Research Council dyspnea scale; CAT = COPD assessment tool; K-BILD = King’s Brief Interstitial Lung Disease questionnaire; Q = question.

Reports of respiratory exacerbations were more frequent among individuals with self-reported COPD (43.5% reporting ≥ 1 exacerbation) then among those at high-risk for COPD (31.4%), while the absence of exacerbations was inherent to the definition of the low-risk group.

### Responses to Questionnaires

**Figure 2.**
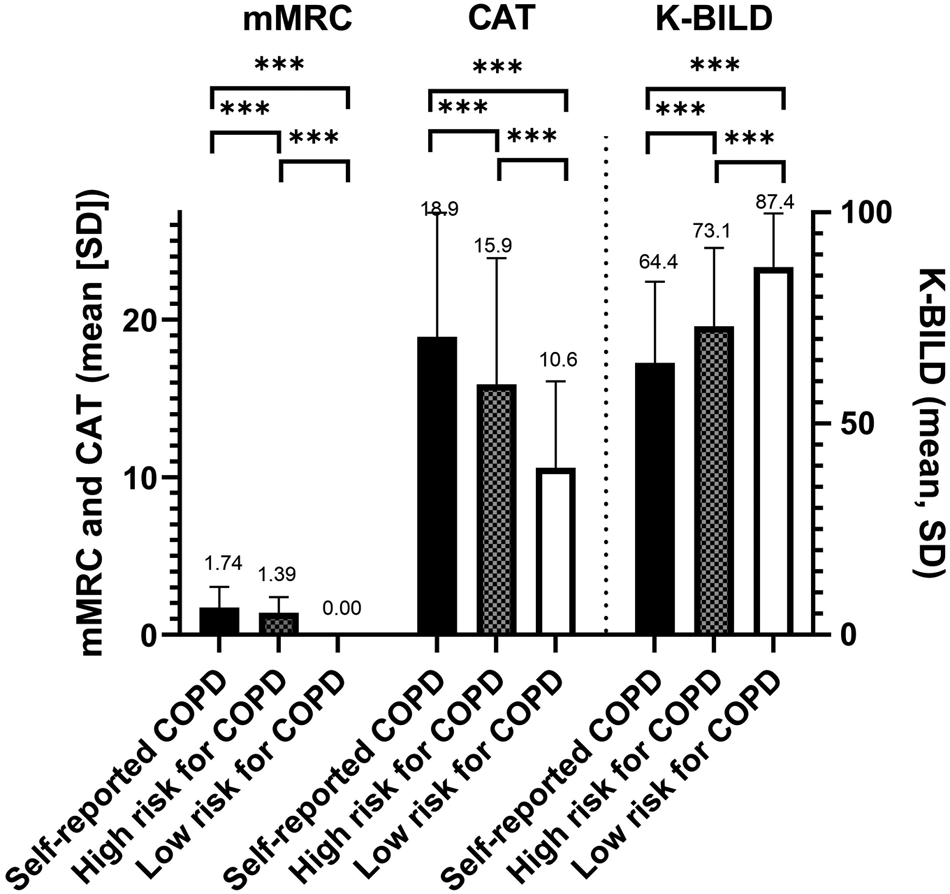
Responses to the Modified Medical Research Council dyspnea scale (mMRC), the COPD assessment tool (CAT), and the King’s Brief Interstitial Lung Disease questionnaire (K-BILD) by self-reported COPD (n = 799), high-risk (n = 6569), and low-risk (n = 1411) for COPD. *P*-values derived from ANCOVA tests adjusted for age, gender, and current smoking status. ***P < .0001.

Greater breathlessness and impairment in HRQoL were seen in people with COPD (mean ± SD mMRC 1.74 ± 1.28, CAT 18.9 ± 7.9, K-BILD 64.4 ± 19.2) than in those at high risk for COPD (1.39 ± 1.0, 15.9 ± 8.0, and 73.1 ± 18.6, respectively; all *P* < .0001; Table 1, Figure 2). The low-risk group scored significantly better (0.0 ± 0.0, 10.6 ± 5.5, and 87.4 ± 12.8, respectively) relative to those with established COPD (all *P* < .0001) or at high risk for COPD (all *P* < .0001).

**Figure 3.**
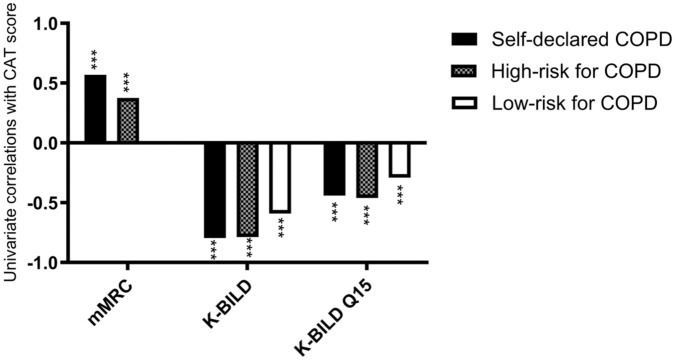
Univariate correlations (Spearman’s correlation coefficients) between the COPD assessment tool (CAT) and the Modified Medical Research Council dyspnea scale (mMRC), the King’s Brief Interstitial Lung Disease questionnaire (K-BILD), and K-BILD item Q15 (“financially worse off because of your lung condition”), by self-reported COPD (n = 799), high-risk for COPD (n = 6569) and low-risk for COPD (n = 1411) groups. ***P < .0001 versus CAT within each category.

A significant positive correlation was observed between CAT and mMRC scores in individuals with self-reported COPD (*r* = .57, *P* < .0001) and in those classified as high risk for COPD (*r* = .38, *P* < .0001), with a numerically stronger association in the self-reported COPD group. In parallel, CAT scores were strongly and consistently negatively correlated with K-BILD scores across groups, indicating that higher symptom burden was associated with poorer health-related quality of life (Figure 3). Notably, the strength of this correlation was numerically not different between the self-reported COPD group and the high-risk for COPD group, relative to the low-risk group. The specific financial implications question (Q15) from K-BILD was negatively correlated with CAT, but only weakly, and was not numerically different across groups.

Across smoking status subgroups (current, former, and never smokers; Table 2), individuals with self-reported COPD who were current smokers exhibited greater impairment in respiratory HRQoL, with higher mMRC and CAT scores and lower K-BILD scores, compared with individuals with self-reported COPD who were former or never smokers.

**Table 2.** Self-reported Characteristics Across COPD Risk Groups in Respondents with Complete Questionnaire Data by Smoking Status.

	Current smokers			Former smokers			Never smokers		
Variables	Self-declared COPD	High-risk for COPD	Low-risk for COPD	Self-declared COPD	High-risk for COPD	Low-risk for COPD	Self-declared COPD	High-risk for COPD	Low-risk for COPD
N	145	2284	483	598	3299	618	56	986	310
Age, years	60.1 (10.3)	56.7 (10.8)	52.5 (10.6)	65.4 (8.9)	60.3 (11.2)	56.3 (11.5)	61.4 (13.4)	55.7 (11.8)	54.5 (11.3)
Sex									
Male (M)	42.1% (61)	33.5% (764)	33.5% (162)	43.0% (257)	38.8% (1280)	44.3% (274)	39.3% (22)	30.2% (298)	43.5% (135)
Female (F)	57.9% (84)	66.5% (1520)	66.5% (321)	57.0% (341)	61.2% (2019)	55.7% (344)	60.7% (34)	69.8% (688)	56.5% (175)
Experienced respiratory exacerbations?									
Never	−57.9% (84)	−73.5% (1678)	−100% (483)	−55.9% (334)	−67.9% (2240)	−100% (618)	−58.9% (33)	−59.7% (589)	−100% (310)
Once	−21.4% (31)	−15.0% (343)	−0% (0)	−18.9% (113)	−17.2% (566)	−0% (0)	−16.1% (9)	−19.4% (191)	−0% (0)
≥Twice	−20.7% (30)	−11.5% (263)	−0% (0)	−25.3% (151)	−14.9% (493)	−0% (0)	−25.0% (14)	−20.9% (206)	−0% (0)
mMRC	1.84 (1.27)	1.44 (0.99)	0 (0)	1.75 (1.28)	1.38 (0.98)	0 (0)	1.29 (1.12)	1.30 (0.94)	0 (0)
CAT	21.3 (8.2)	16.5 (8.1)	11.1 (5.6)	18.5 (7.8)	14.9 (8.0)	9.8 (5.1)	17.7 (8.1)	17.7 (7.4)	11.2 (6.1)
K-BILD	61.9 (18.9)	72.2 (18.7)	86.3 (13.2)	64.5 (19.3)	74.1 (19.0)	88.3 (12.2)	69.6 (18.6)	72.1 (16.8)	87.4 (13.1)
K-BILD Q 15: Are you financially worse off because of your lung condition?	5.1 (1.9)	6.0 (1.6)	6.6 (1.0)	5.4 (1.7)	6.1 (1.4)	6.7 (0.8)	5.8 (1.7)	6.1 (1.4)	6.7 (0.8)

*Note.* Data given as mean (SD) or % (n).

COPD = chronic obstructive pulmonary disease; mMRC = Modified Medical Research Council dyspnea scale; CAT = COPD assessment tool; K-BILD = King’s Brief Interstitial Lung Disease questionnaire; Q = question.

## Discussion

Early identification of COPD is key to preventing poor health outcomes, morbidity, and mortality.^1,2^ The overarching goal of this project using a web-based tool was not to diagnose COPD, but to identify patients who would benefit from further COPD testing. Of the 8779 respondents to the respiratory HRQoL questionnaires, we algorithmically identified 6569 individuals with a high a priori risk of COPD. A key observation was that the tool successfully identified a gradient in respiratory HR between those who self-reported having COPD and those with high- and low-risk for COPD, which underpins the fair likelihood that this approach de facto identifies some with previously undiagnosed COPD. The other notable observations were (1) that a substantial proportion of individuals with self-reported COPD were current smokers, although at a lower prevalence than in the high- and low-risk groups, likely reflecting smoking cessation after diagnosis and selection effects inherent to this web-based survey; and (2) that there is a strong correlation between CAT and K-BILD both in people with self-reported COPD and those with a high risk for COPD, Figure 3.

Large randomized studies in primary care, have demonstrated that systematic, targeted approaches based on symptom questionnaires substantially outperform routine practice in identifying previously undiagnosed COPD. In Target COPD, active case finding using mailed questionnaires followed by spirometry was more than twice as effective as opportunistic case finding and markedly superior to usual care, while also being cost-effective.^8^ While our study differs in design, setting, and endpoints, the observed gradient in breathlessness and respiratory health-related quality of life across algorithm-defined risk groups mirrors the underlying rationale of targeted case finding. A web-based COPD self-assessment may complement primary care case-finding by enabling symptomatic individuals to initiate risk assessment independently of healthcare contact. Further prospective validation and comparison with established strategies are needed before broader implementation.

In the present study we observed that a substantial proportion of respondents to the website had a high COPD risk, which appears to differ from the findings of other surveys.^18^ The survey was not representative of the general population but should be regarded as targeting individuals with respiratory symptoms, as reflected by a mean CAT score of 10.6 in the low-risk group, which is high compared with a healthy population. Recruitment through a patient organization website likely introduced selection bias, as such platforms are more commonly accessed by individuals with chronic conditions or active health concerns who may be more engaged with and attentive to their symptoms. The requirement for complete responses may have further selected for more symptomatic or motivated individuals. Attrition was also partly attributable to the survey design, which allowed users to bypass remaining questionnaire items and access recommendations early, prioritizing usability over complete data capture. Nevertheless, when assessing the background profile of this large cohort (eg, mean age 58.4 years), they do not fall too far off from other studies^19^ and suggest that there is a high degree of respiratory burden in a population who seeks to be tested using a free web-based tool. Primary care physicians should be aware of this and conduct appropriate testing to determine if symptoms are due to COPD or other underlying conditions so that patients can receive the correct treatment, provided that the respondents act on the prompt they were given. Although we do not know the sensitivity or specificity of this approach, it is conceivable that it would identify a large proportion of people with COPD, given the similarity with a US study from October 2018 to April 2022 involving 4325 adults ages 45 to 80, that confirmed that 110 participants (2.5%) of the study sample had undiagnosed moderate to severe forms of COPD, of which the algorithmic questionnaire identified 48%.^9^ However, it also provided false positives for 11% who did not have COPD. Thus, the effectiveness of this approach needs further validation.

Of note, information about the hereditary disorder α-1 antitrypsin deficiency (AATD), appears to be a difficult question, and people are not aware of this in a broader context. AATD remains the most common monogenic risk factor for COPD (homo- or heterozygosity at the *SERPINA1* Z allele).^20^ Homozygosity occurs in approximately 1 in 3000 individuals, however, heterozygosity is more common (approximately 1 in 25), and the increased risk of COPD in this group in smokers has been suggested to be under-recognized.^21^ This aspect may warrant greater attention, particularly given the relatively high prevalence of current smokers observed in this survey, even among those in the high-risk COPD group. Current guidelines recommend testing for α_1_ antitrypsin deficiency in all patients with COPD to ensure an accurate diagnosis and appropriate management.^1^

An interesting observation is the high number of current smokers in the self-reported COPD group. This is higher than that in the general population of Norway, where, according to Statistics Norway 2024, it was 8% in both men and women,^22^ however, this could be due to selection bias.

K-BILD was included in this study to obtain general insight into how it performed compared to other questionnaires in this population of respondents seeking a self-assessed COPD risk evaluation. K-BILD has not been validated in this type of population, and there are therefore multiple limitations to the interpretation of these scores, including the lack of symptomatic evaluation of cough in K-BILD, which is a common clinical feature of COPD.^2^ Nevertheless, it is interesting to note the low average K-BILD score in the self-reported COPD group of 64.4; which is only slightly above what has been reported in people with systemic sclerosis-associated ILD in Norway (63.1).^16^ The strong correlation between CAT and K-BILD indicates that higher symptom burden is consistently associated with poorer overall health-related quality of life, supporting the relevance of symptom-based assessment in this population.

This survey has many limitations, including a lack of control for comorbidities. COPD often occurs in parallel with other conditions with overlapping symptoms, such as heart failure and sleep apnea, which can significantly affect the disease course. On a more principal level, since all responses are anonymous and collected on a website, we are unable to make quality control to ensure that this is actual and real information. Although studies on Internet surveys have been relatively reliable.^23^ There are also limitations for external validity, since people without access to the Internet are excluded (eg, elderly, or people with low access to computer systems), or people with reading difficulties. Finally, the outcomes of the surveys and their recommendations have not been validated, and further studies should explore this aspect in a properly powered manner.

## Conclusion

The early identification of COPD is crucial for preventing adverse health outcomes, including increased morbidity and mortality. An algorithm based on survey responses related to breathlessness and respiratory HRQoL was able to effectively distinguish between individuals at high and low risk of COPD. The high-risk COPD group shared several key characteristics with participants who self-reported a COPD diagnosis, supporting the potential of this web-based tool for identifying individuals with undiagnosed COPD. These findings suggest promise for the use of the tool in early case finding and targeted intervention; however, further validation is required.

## Supplemental Material

sj-docx-1-inq-10.1177_00469580261432827 – Supplemental material for Web-Based COPD Risk Self-Assessment Identifies a High-Risk Group With HRQoL Resembling Self-Reported COPD: Cross-Sectional Survey Supplemental material, sj-docx-1-inq-10.1177_00469580261432827 for Web-Based COPD Risk Self-Assessment Identifies a High-Risk Group With HRQoL Resembling Self-Reported COPD: Cross-Sectional Survey by Bernt Bøgvald Aarli, Kjell Garatun-Tjeldstø, Odd Erik Johansen, Laxmi Bhatta, Arnt-Ove Hovden, Oeystein Naess, Marthe Gundersen and Bjarte Nore in INQUIRY: The Journal of Health Care Organization, Provision, and Financing

sj-docx-2-inq-10.1177_00469580261432827 – Supplemental material for Web-Based COPD Risk Self-Assessment Identifies a High-Risk Group With HRQoL Resembling Self-Reported COPD: Cross-Sectional Survey Supplemental material, sj-docx-2-inq-10.1177_00469580261432827 for Web-Based COPD Risk Self-Assessment Identifies a High-Risk Group With HRQoL Resembling Self-Reported COPD: Cross-Sectional Survey by Bernt Bøgvald Aarli, Kjell Garatun-Tjeldstø, Odd Erik Johansen, Laxmi Bhatta, Arnt-Ove Hovden, Oeystein Naess, Marthe Gundersen and Bjarte Nore in INQUIRY: The Journal of Health Care Organization, Provision, and Financing
