# Supplementary material for: Web-Based COPD Risk Self-Assessment Identifies a High-Risk Group With HRQoL Resembling Self-Reported COPD: Cross-Sectional Survey
Source: Inquiry. 2026 Apr 8;63:00469580261432827. doi: 10.1177/00469580261432827 (PMC13065267; doi:10.1177/00469580261432827)
Supplement: sj-docx-2-inq-10.1177_00469580261432827 – Supplemental material for Web-Based COPD Risk Self-Assessment Identifies a High-Risk Group With HRQoL Resembling Self-Reported COPD: Cross-Sectional Survey [file sj-docx-2-inq-10.1177_00469580261432827.docx]

The Online Questionnaire – English translation

#### **MANUSCRIPT ID: INQ-25-0881**

**MANUSCRIPT TITLE:** **Web-based COPD risk self-assessment identifies a high-risk group with HRQoL resembling self-reported COPD: cross-sectional survey**

**Could I have COPD?**
More than 300,000 people in Norway have COPD without knowing it. Check whether you may be at risk. Take the self-test.

1. Are you older than 35 years? (Yes/No)
2. Have you been significantly exposed to smoke or dust? (Yes/No)
3. Do you have chronic respiratory symptoms? (Yes/No)
4. Have you experienced exacerbations requiring antibiotics, prednisolone, or hospitalization during the past 12 months? (None, once, twice, three or more times)
5. mMRC Questionnaire

**You may have COPD.** Consider booking an appointment with your doctor for further evaluation. If you are diagnosed with COPD, it is possible to live well with the condition. There are several targeted measures available on the recommendations page.

May we ask a few additional questions about your symptoms? (Yes, please / No, thank you)

**Prepare for your medical appointment.** To ensure the best possible treatment, you may enter some additional information about yourself. Anonymity is ensured. No personal identifying information is recorded. (Enter information / Skip to recommendations)

1. Sex (Male/Female)
2. Age
3. Smoking status (Current smoker / Former smoker / Never smoker)
4. COPD Assessment Test (CAT)
5. In some families, an inherited deficiency of the enzyme alpha-1 antitrypsin may occur, which can cause COPD. Have you been tested for this? (Yes, deficiency confirmed / Yes, normal result / No, not tested / Do not know)
6. The K-BILD Questionnaire

**I have COPD**
You can live well with COPD, but it is important to receive appropriate and targeted treatment. Review your treatment.

**Optimal treatment**
To provide advice on the most appropriate treatment, we need to ask you a few questions.
**Inhaled medications:** Please click on the images of the medications you use on the following pages.

1. Have you experienced exacerbations requiring antibiotics, prednisolone, or hospitalization during the past 12 months? (None, once, twice, three or more times)
2. MMRC Questionnaire

Thank you for your answers. You can live well with COPD. It is important to receive the right, targeted treatment. Get an overview on the recommendations page.

May we ask a few additional questions about your symptoms? (Yes, please / No, thank you)

**Review your treatment**

1. Sex (Male/Female)
2. Age
3. Smoking status (Current smoker / Former smoker / Never smoker)
4. COPD Assessment Test (CAT)
5. In some families, an inherited deficiency of the enzyme alpha-1 antitrypsin may occur, which can cause COPD. Have you been tested for this? (Yes, deficiency confirmed / Yes, normal result / No, not tested / Do not know)
6. The K-BILD Questionnaire

**Both pathways ended on an information page providing general guidance, including the following items:**

1. **Medical consultation:**
   A physician can assess lung function using spirometry, which measures how fast and how much air you can exhale. The physician can also provide support with smoking cessation, vaccinations, inhaled medications, nutritional supplements, pulmonary rehabilitation, and referral to a respiratory specialist.
2. **Smoking cessation: (If smoking was reported)**
   Smoking is the most important risk factor for COPD. Smoking cessation slows the decline in lung function, reduces symptom progression, and improves survival in COPD. Support, motivational counseling, guidance, and smoking cessation aids can be provided by a physician.
3. **Medication:**
   Inhaled medications can relieve symptoms, improve functional capacity, and reduce the risk of exacerbations in COPD. Incorrect inhaler technique is common. If specific inhaled medications were selected in the “Review your treatment” pathway, a link to an inhalation technique instruction video for the selected inhaler was provided.
4. **Physical activity:**
   Along with smoking cessation, physical activity is one of the most important and effective interventions for reducing breathlessness at all stages of COPD. Regular exercise is recommended, including endurance, strength, and flexibility training three times per week, with at least two supervised sessions. General practitioners may refer patients to pulmonary rehabilitation. Referral is not required for physiotherapy.
5. **Vaccination:**
   Patients with COPD are recommended to receive annual influenza and COVID-19 vaccinations. Pneumococcal vaccination is recommended for all individuals aged ≥65 years, while younger patients with COPD should undergo an individual assessment of need. Vaccine protection may last up to 10 years. Pneumococcal infections account for approximately 30–50% of pneumonia cases.
6. **LHL (Norwegian Heart and Lung Association):**
   The peer support helpline (“Likepersonlinjen”) complements support from healthcare services, family, and friends. All peer supporters are trained by LHL and are bound by confidentiality. Telephone: +47 22 79 90 90 (Monday–Friday, 10:00–21:00).
   LHL has approximately 250 local chapters across Norway. You can apply for membership via the LHL website. Find your local chapter by entering your postal code: (Field for **postal code)**
